# Inferior turbinectomy: what is the best technique?^☆^

**DOI:** 10.1016/j.bjorl.2017.12.003

**Published:** 2017-12-22

**Authors:** Renato Roithmann

**Affiliations:** a Universidade Luterana do Brasil (ULBRA), Faculdade de Medicina, Departamento de Otorrinolaringologia, Canoas, RS, Brazil; b Mount Sinai Hospital, Department of Otolaryngology, Toronto, Canada

Turbinectomy is a surgical procedure with an excellent outcome for many patients with nasal obstruction resistant to clinical treatment. Surgery on either the inferior or the middle turbinate can result in quite satisfactory results. However, the best surgical approach to the inferior turbinate is a matter for discussion and, to date, there is no gold standard technique that can be applied to all cases. Regardless of technique and equipment, creating more space for the passage of air and minimizing complications are the desired goals.

Procedures range from simple submucosal cauterization associated with lateral fracture to resection, to a greater or lesser extent. The materials and equipment used include very sharp scissors or more sophisticated equipment such as microdebriders and radiofrequency ablation.

There is abundant literature on the subject, but comparative studies among the different approaches are scarce. There are several reasons for this, and the wide variability in nasal anatomy among individuals is undoubtedly one of the factors limiting the choice of a single technique for all cases.

But then, how can one choose the best technique?

It depends on each case. More specifically, it depends on the anatomy of the inferior turbinate (whether the hypertrophy is more related to bone or mucosa), the extent of the hypertrophy (whether it is more anterior or posterior), the response to previous interventions, available equipment, and the surgeon's skill. Another point to be considered is how to preserve the nasal physiology.

The following are some essential clinical and physiological concepts that should be considered by the surgeon:

- Studies show that more intranasal space does not necessarily mean better nasal breathing. All otorhinolaryngologists have seen patients who underwent extensive turbinectomies but still complain of nasal obstruction or even that their obstruction worsened after the surgery.^1^
- The symptom of nasal obstruction is poorly correlated both with rhinoscopy and imaging findings, and with specific nasal permeability tests such as rhinomanometry and acoustic rhinometry. A patient can complain of obstruction and have a normal examination, or the opposite can occur, i.e., the examination is abnormal, and the patient has no complaint.^2^
- Under normal conditions the head of the inferior turbinate in the nasal valve region represents approximately 50% of intranasal airflow resistance. Decreasing the head of the inferior turbinate results in significant increase in airflow.
- The correlation between the nasal area and airflow is exponential. Small increases in area generate large increases in airflow.^2^
- Thermoreceptors present in the nasal vestibular skin and nasal mucosa are also responsible for the sensation of adequate breathing (cold thermoreceptor activation through the trigeminal nerve).^3^

In brief, the choice of surgical technique should take into consideration, in addition to the local anatomy, nasal physiology concepts. Breathing well through the nose requires adequate air space and nasal sensation.^2^, ^3^ The main function of the nose is the modification of inspired air. For that to occur, it is necessary that air enter the nose; however, the presence of viable mucosa and nasal tissues is also vital for this function to occur.

For these reasons, we have developed a relatively simple technique, which we currently call “Five-minute turbinectomy”, due to the mean time it takes to be performed.^4^, ^5^ The technique is based on the turbinoplasty described by Mabry^6^ in 1988, with the difference of using cutting forceps and resecting the inferior turbinate head.

The four essential physiological principles for the approach are:

- The critical zone is the nasal valve region (inferior turbinate head);
- Small area increase = large increase in airflow (exponential correlation between area and airflow);
- Removing more does not mean greater improvement (no correlation between breathing sensation and transnasal airflow area);
- Nasal sensation depends in part on the nasal mucosa of the inferior turbinate.

After local vasoconstriction, the head of the inferior turbinate is resected with cutting forceps. This is the basic difference between this technique and Mabry's turbinoplasty. The next step is the elevation of a medial mucosal flap, of greater or lesser extension according to each clinical situation. Subsequently, the resection of the bony portion and the inferior and lateral mucosa are performed and, finally, the medial flap is repositioned. A comparative study between this technique and the classical partial turbinoplasty with scissors showed similar preliminary outcomes in relation to nasal obstruction relief.^5^

In conclusion, the surgical management of the inferior turbinate should be individualized, according to the patient's clinical situation, and the attending physician should know all available techniques and use them as required. Herein, experience-based medicine seems to be more important than evidence from randomized clinical trials. The surgical techniques used should prioritize improvement in air space and nasal function.

## Conflicts of interest

The authors declare no conflicts of interest.
